# UV-C Irradiation Enhances Antioxidant Capacity and Delays Postharvest Shrinkage of Passion Fruit

**DOI:** 10.3390/foods15142464

**Published:** 2026-07-11

**Authors:** Qunyi Wang, Juan Qin, Xiangbin Xu, Yonggui Pan, Zhengke Zhang, Wanli Zhang, Lanhuan Meng

**Affiliations:** School of Food Science and Engineering, Hainan University, Haikou 570228, China; wqy1030222@126.com (Q.W.); 18276834644@163.com (J.Q.); xbxuibcas@126.com (X.X.); yongui123@126.com (Y.P.); zhangzhengke@hainanu.edu.cn (Z.Z.)

**Keywords:** UV-C, passion fruit, antioxidant system, reactive oxygen species, shelf life

## Abstract

In response to the water loss, shrivelling, and oxidative ageing commonly observed in Golden passion fruit after harvest, this study systematically evaluated the regulatory effects of short-wave ultraviolet (UV-C) treatment. The results showed that UV-C treatment at 3.6 kJ m^−2^ significantly curtailed the increases in weight loss and shrivelling index during storage. Low-field nuclear magnetic resonance (LF-NMR) analysis revealed that this treatment effectively maintained the stability of water distribution in fruit tissues by delaying the conversion of free water to bound water. Regarding antioxidant regulation, UV-C treatment significantly increased the activities of key antioxidant enzymes, including superoxide dismutase (SOD), catalase (CAT), ascorbate peroxidase (APX), and glutathione reductase (GR), while promoting the accumulation of non-enzymatic antioxidants, such as total phenols, total flavonoids, and ascorbic acid (AsA). These changes synergistically enhanced reactive oxygen species (ROS)-scavenging capacity, leading to significant reductions in H_2_O_2_ content and malondialdehyde (MDA) levels, thereby alleviating lipid peroxidation damage to cell membranes. Overall, UV-C treatment effectively maintained cell membrane integrity and regulated water migration through the coordinated regulation of enzymatic and non-enzymatic antioxidant defence systems, thereby delaying passion fruit shrivelling and improving postharvest quality and storage stability.

## 1. Introduction

Passion fruit (*Passiflora edulis* Sims) is native to Brazil and belongs to the genus *Passiflora* within the family Passifloraceae; it is a perennial evergreen climbing vine commonly found in subtropical and tropical regions [1]. Golden passion fruit (*Passiflora edulis* f. *flavicarpa*) is a tropical and subtropical perennial evergreen vine. In recent years, golden passion fruit has been cultivated on a large scale in Chinese provinces such as Yunnan, Guangdong, Guangxi, and Hainan. This fruit is rich in vitamins, amino acids, and essential minerals and is widely favoured by consumers for its abundant nutritional content. Furthermore, passion fruit is rich in various bioactive compounds, such as ascorbic acid (AsA), polyphenols, and flavonoids [2]; these phytochemicals have attracted attention due to their antioxidant capacity and potential health benefits [3]. Additionally, passion fruit and its by-products have been shown to offer potential benefits for the management of chronic conditions such as diabetes and hypertension [4]. However, passion fruit is a climacteric fruit, and the primary postharvest issues affecting it are fruit senescence and water loss-induced shrivelling caused by evaporation [5]. Moreover, during postharvest storage, the fruit is highly susceptible to microbial infection, rotting, and spoilage [6]. These factors reduce fruit quality, induce browning, and decrease commercial value [7]. Consequently, effective postharvest techniques are required to maintain the quality of passion fruit. In recent years, various chemical and non-chemical methods have attracted widespread attention for preventing the deterioration of passion fruit. Examples include CaCl_2_ treatment [8], bio-based composite coatings [9,10], 1-MCP [11], exogenous melatonin [12], and controlled atmosphere packaging [13]. However, some of these methods carry potential risks, such as high implementation costs in commercial settings, limited ability to control microbial spoilage, and the potential for uneven localised discolouration [14], which may affect the fruit’s aromatic compounds. With the continued growth in global demand for passion fruit, postharvest preservation and shelf-life extension technologies hold significant market value. At the same time, as the concept of green consumption becomes more widespread, consumers are increasingly inclined to choose fresh fruit that has not undergone chemical treatment and is preserved using green methods.

Ultraviolet (UV) refers to invisible light with a wavelength range of 100–400 nm. Depending on the wavelength range, UV radiation is primarily divided into three categories: UV-A, UV-B, and UV-C. UV-C, also known as germicidal UV light, is a residue-free, non-thermal, and non-chemical physical preservation method. Treatment with UV-C (100–280 nm) can effectively improve the quality of various crops and extend their shelf life. As a non-toxic, pollution-free, residue-free, safe, and effective non-chemical preservation technology, it has been widely applied in the postharvest preservation of various fruits and vegetables, and has been shown to reduce storage rot in numerous vegetable crops [15]. The antibacterial mechanism of UV-C lies in the absorption of its photons by conjugated carbon–carbon double bonds in proteins and nucleic acids. This action induces structural changes in DNA and RNA, thereby disrupting key processes such as replication and transcription, ultimately inhibiting the proliferation of microorganisms such as viruses, bacteria, and moulds, leading to their inactivation [16]. UV-C has been demonstrated to be effective in strawberries [17], mangoes [18], pears [19], peaches [20], grapes [21], and tomatoes [22], and to extend their postharvest shelf life. However, in addition to its direct bactericidal effect, UV-C can also stimulate plant defence mechanisms. Low doses of UV-C stimulate beneficial responses in biological materials, a phenomenon known as ‘hormesis’ [23]. In addition, relatively low doses are required to induce this hormetic effect; therefore, it is necessary to select an appropriate and effective UV-C treatment dose for practical applications. Low-dose UV-C treatment has been shown to induce disease resistance in postharvest fruit; a UV-C dose of 2.0 kJ m^−2^ preserved the commercial characteristics and firmness of guavas, reduced weight loss, effectively increased the content of bioactive compounds such as total phenolics and total flavonoids, and upregulated antioxidant enzyme activity [16]. In the preservation of pepino fruit, UV-C treatment at 1.5 kJ m^−2^ significantly enhanced antioxidant enzyme activity and effectively maintained the overall levels of acids, aldehydes, and esters in the fruit [24]. Several studies have also highlighted the role of low-dose UV-C treatment in the preservation of fruit and vegetables by delaying cellular senescence and reducing damage to cellular structures [25], inhibiting quality deterioration and flavour loss [26], and regulating physiological metabolism to stimulate the fruit’s antioxidant system [27], thereby extending the shelf life of the fruit.

The delay in fruit ripening induced by UV-C treatment may be attributed to the involvement of other defence mechanisms, such as the induction of antioxidant systems. As an abiotic stressor, UV-C can effectively stimulate the antioxidant systems of postharvest fruit, including both enzymatic and non-enzymatic antioxidant systems. Passion fruit peel and pulp are rich in various antioxidants, including AsA, flavonoids, and phenolic compounds [28], whilst antioxidant enzymes such as superoxide dismutase (SOD), catalase (CAT), glutathione reductase (GR), and ascorbate peroxidase (APX) constitute its enzymatic defence system. Disruption of reactive oxygen species (ROS) metabolism is one of the primary causes of accelerated disease development in postharvest fruit; enhancing the antioxidant system may improve the fruit’s resistance to disease [29]. The gradual imbalance of the antioxidant system during postharvest senescence is a major cause of quality deterioration; therefore, delaying postharvest senescence by regulating ROS metabolism and antioxidant enzyme activity is of significant importance. Plants typically accumulate secondary metabolites in response to abiotic stress or stimulation by bioinducers. As the primary function of plant secondary metabolites is to protect plants from biotic and abiotic stresses, controlling UV-C treatment of postharvest fruit can increase the content of these secondary metabolites [30]. Research by Liu et al. [31] demonstrated that when tomatoes were irradiated with 4 kJ m^−2^ of UV-C, the phenylpropanoid pathway in the fruit was likely activated following UV-C exposure, promoting the accumulation of total phenolics, phenolic acids, and flavonoids within the fruit, and thereby improving the antioxidant capacity of the tomatoes postharvest.

To date, there have been no studies examining the effects of UV-C treatment on physiological changes in passion fruit following harvest. Furthermore, although the application of UV-C in the postharvest preservation of fruit and vegetables has attracted attention, systematic research into its role in regulating antioxidant activity in passion fruit remains lacking. Consequently, this study used Golden passion fruit as the experimental material to analyse the effects of UV-C treatment on ROS accumulation and the antioxidant system, including changes in AsA and phenolic compound content, as well as the activities of SOD, CAT, APX, and GR. Furthermore, the regulatory effects on oxidative damage and cell membrane stability were assessed by evaluating H_2_O_2_ and MDA levels. Concurrently, a comprehensive evaluation of the efficacy of UV-C treatment in delaying postharvest senescence was conducted in conjunction with changes in fruit quality.

## 2. Materials and Methods

### 2.1. Fruit Material and Treatment

In this study, the yellow passion fruit variety ‘Qinmi 9′ was obtained from a passion fruit plantation in Fushan Town, Chengmai County, Hainan Province, and transported to the laboratory in Haikou City within 24 h. Fruits from the same batch were carefully selected based on edible quality, similar ripeness (80–90%), uniform weight (80 ± 10 g), oval shape, longitudinal diameter (60 ± 5 mm), transverse diameter (55 ± 5 mm), and the absence of mechanical damage. The selected fruits were immersed in 0.1% (*v*/*v*) sodium hypochlorite for 5 min, rinsed with distilled water, and air-dried.

The selected fresh passion fruits were then divided into two groups, with each group containing 50 fruits. The first group served as the control group (CK) and did not undergo UV-C irradiation; the second group was the UV-C-treated group, which was irradiated for 20 min in a custom-built, enclosed, light-tight UV-C light box. The UV-C treatment apparatus comprised a light-tight chamber measuring 35 cm × 40 cm × 35 cm, which had been constructed domestically. The UV-C light source comprised a T5 UV-C lamp (Philips) with a primary emission wavelength of 254 nm; the lamp was mounted at the top of the chamber. During the treatment process, the fruits were meticulously arranged in a single, uniform layer at the base of the chamber. A distance of 30 cm was maintained between the fruit surfaces and the light source to minimise mutual shading between fruits and enhance the uniformity of irradiation. The UV-C irradiance at the fruit surface was 3 W m^−2^. The UV-C irradiation dose was calculated using the following formula: ‘dose = irradiance × exposure time’. In the context of this study, a 20 min UV-C treatment was found to correspond to an irradiation dose of 3.6 kJ m^−2^. The fruit was manually rotated once at the 10 min mark during the irradiation process to ensure uniform exposure of the fruit surface to ultraviolet light. Temperature changes within the chamber were monitored throughout the treatment to prevent temperature rises during UV-C irradiation from interfering with the experimental results. The control group of fruits was maintained under identical environmental conditions for the same duration, but did not receive UV-C irradiation. Prior to the main experiment, a preliminary screening experiment on UV-C treatment doses was conducted to assess the effects of different irradiation durations on the postharvest quality of passion fruit. In the preliminary experiment, passion fruit were subjected to UV-C irradiation treatments of 5, 10, 20 and 40 min, respectively. In light of the observed fluctuations in weight loss rate, shrivelling index and appearance quality across the various treatment groups during the preliminary experiment, a 20 min UV-C treatment—corresponding to 3.6 kJ m^−2^—was designated as the treatment dose for the subsequent formal experiment.

After treatment, all fruits were placed in plastic, breathable storage boxes and stored at 25 ± 1 °C and 70 ± 5% relative humidity (RH) for 16 days. Samples were collected at the start of storage and at 4-day intervals to evaluate the nutritional value and physiological changes in passion fruit during postharvest storage. Subsequent to the administration of UV-C treatment, samples were collected from both the CK and treatment groups at four-day intervals over the storage period. Flesh or peel samples intended for physiological and biochemical analysis were rapidly frozen in liquid nitrogen and stored at −80 °C. Prior to measurement, frozen samples from the same treatment and sampling time point were thoroughly mixed to form a composite sample for subsequent analysis, with three replicate measurements performed.

### 2.2. Changes in Fruit Appearance, Shrinkage Index, and Weight Loss

The classification of fruits is based on the degree of surface shrivelling, with the shrivelled area expressed as a percentage of the total volume. The following grades are employed: 0 indicates no shrivelling, 1 indicates 0–25% shrivelling, 2 indicates 25–50% shrivelling, 3 indicates 50–75% shrivelling, and 4 indicates 75–100% shrivelling. The shrinkage index is calculated as follows:

$$\mathrm{Shrinkage}\;\mathrm{index}\;=\;\frac{\sum (I\;\times \;N_{i}\;)}{N}\times 100$$

*I*—shrivelling grade, 0–4; *Ni*—number of fruits in that grade, 0–N; and *N*—total number of fruits.

The fruit must be weighed using an electronic analytical balance to determine its mass, and the rate of weight loss must be calculated based on the difference between the mass measured at each stage and the initial mass. The formula is as follows:

$$\mathrm{Weight}\;\mathrm{loss}\;\mathrm{rate}\;=\;\frac{\mathrm{Wo}-\mathrm{Wt}}{\mathrm{Wo}}$$

*Wo*—initial mass, g; *Wt*—mass on the day of measurement, g.

Six fruits were randomly selected from each treatment as fixed samples; the shrinkage index was calculated, and weight loss was determined through continuous weighing during the storage period.

### 2.3. Measurement of Texture Properties

Fruits without mechanical damage or signs of decay were selected and positioned on the designated test platform. Fruit firmness was measured using a texture analyser (TA-XT plus, Stable Micro Systems, Godalming, UK) equipped with a P/2 needle probe. A small section of peel was removed at two opposite equatorial positions prior to measurement. Firmness was determined by a puncture test and expressed in newtons (N). In addition, texture profile analysis (TPA) was conducted using a P/36R cylindrical probe. Fruits without mechanical damage were subjected to a double-compression test, with pre-test, test, and post-test speeds of 1 mm s^−1^, a compression deformation of 15%, a trigger force of 5 g, and a 4 s interval between compressions. Parameters, including springiness, gumminess, resilience, and chewiness, were obtained from the TPA curves [32]. Three fruits were randomly selected each time; two points on opposite sides of the equator were measured for each fruit, and the average value was taken as the texture value for that fruit.

### 2.4. Determination of Chromaticity

A portable colourimeter was utilised to ascertain the colour of passion fruit. This was achieved by measuring L* (lightness), a* (red-green component) and b* (yellow-blue component). Prior to measurement, the colourimeter was calibrated using a standard white calibration plate. At each designated time interval, three fruit samples were randomly selected from each treatment. Colour measurements were taken at four evenly distributed positions around the equatorial region of the peel for each fruit. Due to the occurrence of unevenness in the peel colour during the storage process, a comprehensive measurement of multiple surface positions was undertaken with the objective of mitigating positional variation and thereby attaining a representative value. The mean value of the four measurements was then employed as the colour value for each fruit.

### 2.5. Determination of Titratable Acidity

The titratable acidity (TA) of the samples was measured by neutralizing the supernatant with 0.1 mol L^−1^ NaOH until the pH reached 8.1 ± 0.1, with results expressed as lactic acid percentage [33].

### 2.6. Determination of Soluble Solid Content

The SW35T/53T digital handheld refractometer was used to measure the soluble solid content, expressed as a percentage.

### 2.7. Measurement of LF-NMR

The distribution and migration characteristics of water in passion fruit tissue were analysed using low-field nuclear magnetic resonance (LF-NMR). The fruit samples were sectioned to the required dimensions and placed into NMR sample tubes, which had a diameter of approximately 20 mm. Three fruits were randomly selected each time, and each fruit was measured individually. These samples were then positioned within the NMR spectrometer probe for analysis. The Carr–Purcell–Meiboom–Gill (CPMG) pulse sequence was utilised to acquire relaxation decay signals, thereby obtaining the transverse relaxation data of the samples. Subsequently, the relaxation decay curves were fitted using multi-exponential inversion to derive the T_2_ relaxation time distribution spectrum. It is widely acknowledged that the water content in fruit is classified into three states based on different relaxation time intervals [34]. The three states are bound water (T_21_, 0.1–10 ms), immobilized water (T_22_, 10–100 ms), and free water (T_23_, 100–1000 ms). Of these, bound water is tightly bound to cell wall components and is not easily mobile; immobilized water is primarily present in restricted spaces within the cellular structure; and free water is distributed in larger pores within the tissue and exhibits high mobility. By analysing changes in the areas of the respective relaxation peaks, the internal water state and migration patterns within passion fruit during storage were characterised [35]. Furthermore, NMImagin Ver4.0software for LF-NMR analysers was utilised to generate pseudo-colour maps of water distribution.

### 2.8. Determination of Respiration Rate and Ethylene Production Rate

The method of Adhikary et al. [36] was employed to measure CO_2_ production (mg kg^−1^ h^−1^). This was accomplished using a portable infrared gas analyser (GXH-3010E, Beijing, China) and subsequently adjusted according to fruit mass. The measurements were obtained by placing golden passion fruits in a respiration chamber and calculating the change in CO_2_ concentration per unit time. Measurements were taken at 4-day intervals during the postharvest storage period, with the results expressed as mg kg^−1^ h^−1^. Two randomly selected fruits were weighed and placed in a 1.3 L sealed plastic container as a group. Three groups were sampled each time and sealed at room temperature (25 °C) for 0.5 h. The measurements were performed using a portable infrared headspace analyser, and the sampling probe was subsequently inserted into the container to measure the CO_2_ content.

The ethylene production rate was determined as follows: A single whole passion fruit was placed in a sealed 0.5 L container and kept at ambient temperature (25 ± 1 °C) for 1 h. A 7890A gas chromatograph (Agilent Technologies, Beijing, China) was utilised to detect ethylene released by the passion fruit during storage. The ethylene production rate was calculated based on the peak area. Gas was extracted from the container using a syringe; 0.1 mL of gas was injected into the gas chromatograph each time, and the measurement was repeated three times. The chromatographic conditions were as follows: a GAS-PRO 30 m × 0.32 mm capillary column was utilised, with nitrogen (99.99%) serving as the carrier gas. The column temperature was set at 90 °C, and the injector temperature was adjusted accordingly. The detector temperature was set at 250 °C, and the sample was exposed at 150 °C, expressed as µL kg^−1^ h^−1^. The ethylene production rate was calculated according to the method reported by Guo et al. [37].

### 2.9. Measurement of AsA

The AsA content of fruit was determined by means of the 2,6-dichlorophenol-indophenol titration method, with the results expressed in milligrams per 100 g. A quantity of 10 g of fruit juice sample was weighed, and 2% oxalic acid solution was added to it to reach a final volume of 100 mL. The mixture was then thoroughly agitated, and 5 mL of the extract was titrated with a 2,6-dichloroindophenol standard solution [38].

### 2.10. Total Phenolic Content and Total Flavonoid Content

In accordance with the method described by Tao et al. [39], 1 g of fruit peel tissue was weighed, and 9 mL of pre-chilled 80% (*v*/*v*) methanol solution was added. The mixture was homogenised in an ice bath and then centrifuged at 10,000× *g* for 10 min at 4 °C. Following centrifugation, the extract was stored at 4 °C. The total phenolic content was determined using the Folin–Ciocalteu (FC) colourimetric method. An appropriate volume of the extract was reacted with Folin–Ciocalteu reagent, followed by the addition of sodium carbonate solution. The mixture was then incubated in the dark for a specified period. Following this, the absorbance of the mixture was measured at 760 nm. The results were calculated using a standard curve based on gallic acid and expressed as mg g^−1^.

The total flavonoid content was determined using the aluminium salt colourimetric method. The extract was reacted sequentially with NaNO_2_, AlCl_3_, and NaOH, resulting in the formation of a stable complex. The absorbance of the resulting complex was then measured at 500 nm. The results were expressed as rutin equivalents, with the concentrations expressed as mg g^−1^.

### 2.11. Measurement of Antioxidant Capacity

The 2,2-diphenyl-1-picrylhydrazyl (DPPH) radical scavenging capacity is utilised to evaluate the total antioxidant activity. A 0.2 mM DPPH stock solution was prepared by the method of dilution in anhydrous ethanol. Following thorough mixing of the reaction system, it was subjected to an incubation period of 30 min at room temperature under light conditions. The following parameters were measured: The following solutions were utilised: A_0_ (2.0 mL DPPH stock solution and 2.0 mL 70% ethanol), A_1_ (2.0 mL DPPH stock solution and 2.0 mL extract), and A_2_ (2.0 mL 70% ethanol and 2.0 mL extract) [40]. The scavenging rate was calculated using the following formula:

Scavenging rate (%) = [(A_0_ − A_1_ + A_2_)/A_0_] × 100%.

2,2′-azinobis (3-ethylbenzothiazoline-6-sulfonic acid) (ABTS) radical scavenging capacity was utilised as a metric for the evaluation of antioxidant activity. A quantity of 1.0 g of fruit peel sample was extracted using a 40% ethanol solution, and then subjected to ultrasonic treatment to promote the release of active compounds. The ABTS^+^ working solution was prepared by mixing a 7.0 mmol/L ABTS solution with a 2.5 mmol/L potassium persulfate solution, followed by a reaction for 12 h at room temperature in the dark. The sample extract was then mixed with the ABTS^+^ working solution. Following a reaction time of approximately 4 min, the absorbances were measured at 734 nm. The antioxidant activity was then evaluated based on the radical scavenging rate.

### 2.12. Measurement of Malondialdehyde (MDA) Content, H_2_O_2_ Content

Measure MDA content according to a published method [41]. Homogenise 2 g of passion fruit with 10 mL of 10% trichloroacetic acid (*w*/*v*) in an ice bath, then centrifuge at 10,000× *g* for 20 min at 4 °C. Mix 2 mL of the supernatant with 2 mL of 0.67% thiobarbituric acid (*w*/*v*), boil in a water bath for 20 min, cool, and centrifuge again. Measure the absorbance of the supernatant at wavelengths of 450 nm, 532 nm and 600 nm; results are expressed in μmol·g^−1^.

Determine the H_2_O_2_ content using an H_2_O_2_ content assay kit (Suzhou Grace Biotechnology Co., Ltd., Suzhou, China). Follow the relevant instructions and express the H_2_O_2_ content as μmol·g^−1^.

### 2.13. Antioxidant Enzyme Activities: CAT, SOD, GR and APX

To extract SOD, 0.1 g of fruit peel tissue was added to 2 mL of pre-chilled 0.1 mol L^−1^ potassium phosphate buffer (pH 7.5) and homogenised using a mortar and pestle. The mixture was then centrifuged at 10,000× *g* for 20 min at 4 °C. The supernatant was collected as the crude enzyme extract for SOD activity determination. Following the method described by Zhang et al. [32], SOD activity was determined based on the inhibition of nitroblue tetrazolium (NBT) reduction. One unit of SOD activity was defined as the amount of enzyme required to inhibit NBT reduction by 50%, and the results were expressed as U min^−1^ g^−1^ FW.

CAT, APX, and GR activities were measured using commercial enzyme activity kits (Suzhou Grace Biotechnology Co., Ltd., Suzhou, China). For enzyme extraction, fresh fruit peel tissue was homogenised in the corresponding pre-chilled extraction buffer under liquid nitrogen and centrifuged at 12,000× *g* for 15 min at 4 °C. The supernatant was collected for enzyme activity assays according to the manufacturer’s instructions.

### 2.14. Sensory Evaluation

Sensory evaluation was performed following a previously reported method with minor modifications. A trained panel consisting of eight assessors evaluated the sensory quality of passion fruit during storage. At each sampling interval, fruit samples from both the CK and UV-C treated groups were randomly selected and presented to the panellists in a randomized order. Six sensory attributes were evaluated: overall appearance, peel colour and gloss, absence of browning/speckling, aroma acceptability, eating texture (palatability), and overall acceptability (marketability). These attributes were scored using a 5-point hedonic scale, where 5 represented excellent quality; 4, good; 3, acceptable (the limit of marketability); 2, poor; and 1, unacceptable. All indices were scored positively, with higher scores indicating superior sensory quality. The marketable shelf life was defined as the storage period during which the overall acceptability (marketability) score remained 3, and no severe decay, juice leakage, or strong off-odor was observed. Fruit was considered to have lost its commercial acceptability when the score dropped below 3.

Aroma assessment initially focused on the intact fruit, followed by the evaluation of the pulp aroma upon cutting the sample. The eating texture was rated based on flavor, sugar-acid balance, juiciness, and mouthfeel. For safety reasons, samples exhibiting apparent decay, severe juice leakage, or strong off-odors were excluded from gustatory evaluation. Drinking water was provided for palate cleansing between sample sessions.

### 2.15. Statistical Analysis

The data processing and statistical analyses were performed using Excel 2021 and IBM SPSS Statistics 27. The preparation of the figures was undertaken using Origin 2021 and Adobe Photoshop 2024(PS) software. All data are presented as mean ± standard deviation. Differences between the two groups at the same storage time were analysed using an independent-samples t-test, with significance set at *p* < 0.05. Different letters indicate significant differences between the CK and UV-C-treated groups at the same storage time. Different letters were used to denote which groups showed significant differences. Principal component analysis (PCA) and Pearson correlation analysis were employed to elucidate the multivariate relationships between physiological parameters. The Pearson correlation coefficient was utilised to ascertain the magnitude of the observed correlation. Significance thresholds were demarcated as outlined below: It is evident that the *p*-value is less than 0.05 in the first instance, and less than 0.01 in the second.

## 3. Results and Discussion

### 3.1. Effects of Different UV-C Doses on Shrivelling Index, Weight Loss, and Fruit Appearance of Passion Fruit

**Figure 1:**
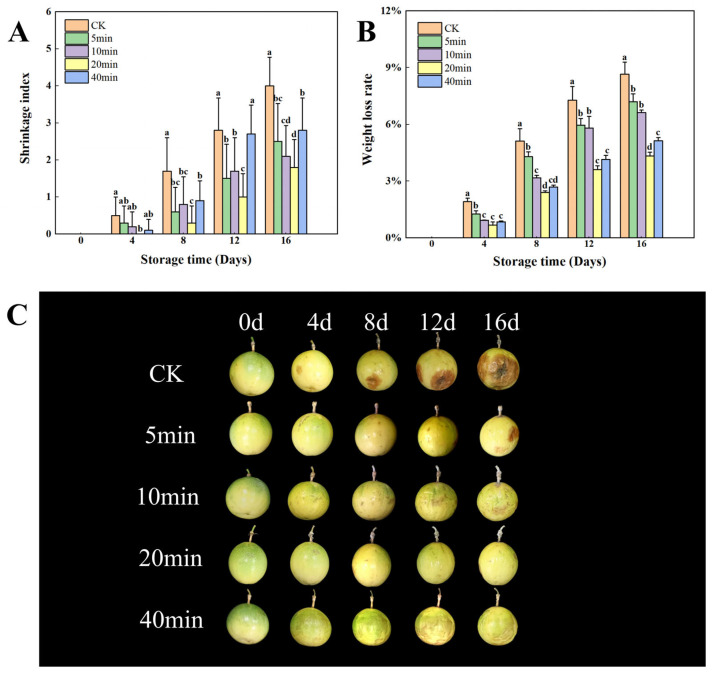
Effects of different UV-C treatment durations on passion fruit during storage. CK represents untreated fruit, while the treatment groups were exposed to UV-C for 5, 10, 20, and 40 min. (**A**) shrinkage index; (**B**) Weight loss; (**C**) Changes in fruit appearance. Error bars represent the standard deviation of the mean. Different letters indicate significant differences among treatments according to the least significant difference (LSD) test (*p* < 0.05).

The shrivelling index is an important indicator of passion fruit quality, reflecting the progression of postharvest shrivelling and quality deterioration. As storage time increased, skin shrivelling became more severe, thereby affecting fruit appearance and commercial quality. As shown in Figure 1A, the shrivelling index of passion fruit increased in all groups, indicating progressive water loss and postharvest deterioration. During storage, the shrivelling index of the CK increased markedly from day 8 and reached 4.0 by day 16, with shrivelled areas covering approximately 80% of the fruit surface. In contrast, the shrivelling indices of all UV-C-treated groups remained lower than that of the CK during the middle and late stages of storage. By the end of the 16-day storage period, the shrivelling indices of all UV-C-treated groups remained below 2.8, which was significantly lower than that of the CK. This finding indicates that UV-C treatment effectively inhibited surface shrinkage in passion fruit. The effectiveness of UV-C treatment in inhibiting passion fruit shrivelling was found to vary depending on the duration of irradiation. While the 5 min and 10 min treatments demonstrated a certain degree of reduction in the fruit shrivelling index, the delaying effect was relatively limited. Notably, the 40 min treatment group maintained a lower level of fruit shrivelling compared to the control group during the later stages of storage. However, the fruit exhibited a certain degree of abnormal browning and uneven colour change. In contrast, the 20 min treatment group exhibited a low shrivelling index throughout the entire storage period, with only noticeable skin shrivelling becoming apparent after the eighth day. As the storage period drew to a close, the degree of shrivelling exhibited by the 20 min treatment group remained significantly lower than that observed in the other treatment groups. This finding suggests that the 20 min treatment is effective in inhibiting postharvest shrivelling in passion fruit.

Weight loss in passion fruit is mainly attributed to substrate consumption and water loss caused by respiration and transpiration [42]. As shown in Figure 1B, weight loss increased continuously during storage in both CK and UV-C-treated fruits. After 16 days of storage, the CK showed a weight loss of 8.66%, whereas weight loss was significantly reduced in all UV-C-treated groups. The weight loss rate of the CK was significantly higher than that of the UV-C-treated groups throughout storage. Specifically, the weight loss of the 20 min treatment group was only 4.34% on day 16, which was comparable to that of the CK on day 8. These results indicate that UV-C treatment effectively mitigated weight loss in passion fruit during storage. This effect may be attributed to a mild stress response induced by UV-C treatment, which activates defence mechanisms, including cell wall reinforcement and reduced membrane permeability. Consequently, UV-C treatment may enhance the structural integrity of the fruit surface and contribute to reduced water loss during storage [43]. In addition, brown spots on the surface of passion fruit are typical postharvest pathological symptoms [44]. The reduction in microbial populations has been shown to help minimise weight loss in treated samples [45]. This may be related to the antibacterial action of UV-C, which limits microbial respiration and cellular degradation, thereby reducing water loss associated with spoilage. These results are consistent with the observed changes in fruit appearance.

Fruit appearance is one of the key indicators used to assess the commercial and nutritional value of passion fruit. As shown in Figure 1C, untreated passion fruit exhibited a marked decline in visual quality during storage, mainly manifested as surface browning and overall shrivelling. At the beginning of storage, the fruits had a smooth and glossy surface with a tight peel structure. However, by day 8, untreated fruits showed visible signs of shrivelling and browning. As storage progressed, the degree of shrivelling in the CK became significantly greater than that in the UV-C-treated groups, and distinct brown spots and lesions appeared on the fruit surface. By the end of storage, severe lesions had developed in the CK, making the fruit unsuitable for commercial sale. The 5 and 10 min treatments delayed the onset of fruit shrivelling and surface browning; however, varying degrees of quality deterioration were still observed during the later stages of storage. Notably, the 20 min treatment promoted the transition of the fruit surface from green to a uniform golden yellow during storage. Although slight shrivelling occurred, the fruits retained a relatively intact appearance and bright colour on days 12 and 16. Conversely, fruits treated for 40 min exhibited obvious browning and substantial colour inconsistency. This phenomenon may be attributed to excessive UV-C irradiation, which can cause surface damage to the fruit. The high-energy photons of UV-C radiation may directly disrupt the cuticle and cell walls, leading to surface oxidation and triggering chlorophyll degradation or phenolic compound oxidation [46]. Previous studies have reported that extending the UV-C irradiation time by 10 min beyond the optimal duration caused severe browning in fresh-cut cabbage leaves [47]. Similarly, excessive UV-C irradiation increased weight loss and wilting symptoms in cucumbers [48]. Therefore, a 20 min irradiation duration was considered effective for delaying postharvest shrivelling and quality deterioration in passion fruit. The subsequent experiments were conducted using a UV-C irradiation dose of 3.6 kJ m^−2^; hereafter, “UV-C treatment” refers to this optimal dose.

It can be concluded from the combined results of the shrivelling index, weight loss rate and changes in appearance that a 20 min UV-C treatment was the most consistent in delaying water loss, shrivelling and deterioration in appearance in passion fruit. The 5 min and 10 min treatments were characterised by relatively low intensity and limited preservative effects. Conversely, the 40 min treatment was observed to potentially induce damage to the fruit peel surface or abnormal discolouration, attributable to the extended irradiation duration. Therefore, under the experimental conditions of this study, a 20 min UV-C treatment (equivalent to 3.6 kJ m^−2^) was determined to be the optimal dosage for subsequent experiments. Apart from the dosage screening experiment, all subsequent analyses regarding the postharvest quality, physiological metabolism, and antioxidant properties of passion fruit were conducted using this UV-C treatment dosage.

### 3.2. Effects of UV-C Treatment on Fruit Colour, Texture, Total Soluble Solids, Consumption of Substrates, and Water Migration

**Figure 2:**
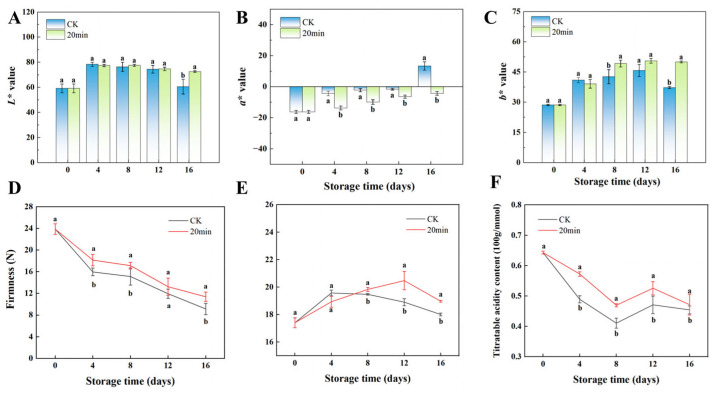
Effects of UV-C treatment on physicochemical properties of passion fruit during storage. CK represents untreated fruit, while the treated group was exposed to UV-C for 20 min. (**A**–**C**) Changes in colour parameters, including L*, a*, and b* values; (**D**) firmness; (**E**) soluble solid content; (**F**) titratable acidity. Error bars represent the standard deviation of the mean. Different letters indicate significant differences between treatments at the same storage time (*p* < 0.05).

Peel colour is one of the key factors influencing consumer choice, and colour intensity reflects changes in fruit appearance and commercial value [49]. As shown in Figure 2A–C, passion fruit displayed a glossy green colour during the early postharvest stage and gradually turned yellow with the onset of the respiratory climacteric. Fully mature fruit exhibited a golden-yellow colour, which is an important feature of the natural ripening process of passion fruit and contributes to its visual appeal and commercial value [50].

During storage, the L* value of passion fruit generally increased initially and then declined. The L* value of the CK decreased significantly during the later stage of storage, indicating reduced brightness in untreated fruit, which was consistent with the visual observations. Huang et al. [51] reported that a continuous decline in brightness was associated with browning during lychee storage. In contrast, UV-C-treated passion fruit maintained a higher brightness level during the later stage of storage. On day 16, the L* value of the UV-C-treated group was 1.34 times that of the CK, indicating better maintenance of fruit appearance and commercial quality.

The a* value remained negative during the early stage of storage, indicating that green colour dominated the fruit surface immediately after harvest. As storage progressed, the passion fruit peel gradually turned yellow, and the a* value shifted towards neutral values, suggesting a gradual fading of greenness. This colour change in golden passion fruit may be related to the rapid degradation of chlorophyll molecules in the peel during ripening [9]. In the CK, the a* value became positive on day 16, increasing from −1.63 on day 12 to 13.35 on day 16, indicating a tendency towards reddening during the late storage period. This phenomenon may be associated with physical deterioration and surface browning of untreated fruit. In contrast, the a* values of UV-C-treated passion fruit remained relatively stable throughout storage, suggesting that UV-C treatment delayed the progression of fruit browning.

During storage, the b* values of both the CK and UV-C-treated groups showed an overall increasing trend, indicating that yellowness gradually increased during ripening. However, the b* value of the CK fluctuated markedly and decreased during the later stage of storage, whereas that of the UV-C-treated group remained relatively high. This finding suggests that UV-C treatment delayed fruit deterioration and shrivelling without interfering with the normal colour transition of passion fruit. After the respiratory climacteric, the b* value of the UV-C-treated fruit remained relatively stable, and the fruit maintained a predominantly golden-yellow colour, thereby contributing to the maintenance of postharvest commercial value.

Therefore, during the early stage of storage, UV-C treatment slowed the yellowing process of passion fruit compared with the CK, while maintaining a higher brightness level. UV-C treatment delayed shrivelling and quality deterioration in passion fruit, thereby preserving visual quality and commercial value. Similar results were reported by Pristijono et al. [52], who found that UV-C treatment significantly delayed the colour transition from green to yellow in mangoes during storage, extended the postharvest life of unripe mangoes, and did not negatively affect certain quality characteristics even under low ethylene concentrations. Similarly, Khubone and Mditshwa [53] reported that an appropriate dose of UV-C irradiation delayed colour development and ripening in tomatoes without adversely affecting fruit quality.

Firmness is closely related to the postharvest quality and storability of fruits and vegetables and reflects changes in fruit ripening and quality deterioration to some extent. As shown in Figure 2D, passion fruit firmness decreased continuously during storage. The CK exhibited a rapid decline in firmness, with a reduction of 33.88% during the early storage period from 0 to 4 days. In contrast, the UV-C-treated group showed a smaller decrease of 23.28%, which was significantly lower than that of the CK. As storage progressed, the difference in firmness loss between the two groups became more pronounced. During the middle and late stages of storage from 8 to 16 days, the UV-C-treated group maintained relatively high tissue strength. By the end of storage on day 16, the firmness of the CK had decreased to 8.92 N, whereas the UV-C-treated group retained significantly higher firmness, indicating that UV-C treatment delayed firmness loss.

Pombo et al. [54] reported that UV-C treatment regulated genes involved in cell wall degradation in stored strawberries, thereby inhibiting cell wall breakdown and maintaining higher firmness. The results of the present study are consistent with the observed changes in water loss. The decline in fruit firmness may be attributed to peel cell wall degradation and changes in tissue structure, which reduce the water-holding capacity of the fruit and lead to firmness loss. Compared with the CK, the UV-C-treated group maintained higher firmness during the middle and late stages of storage, particularly on day 16, indicating that UV-C treatment delayed firmness loss in passion fruit. This effect may be associated with the UV-C-induced inhibition of ethylene release, which helps maintain higher levels of cell wall polysaccharides and tissue strength, thereby delaying fruit shrivelling and softening [55].

In addition to firmness, other textural parameters also changed during storage(Table 1). As storage time increased, fruit springiness gradually declined in both groups, although the difference between the UV-C-treated group and the CK was not statistically significant. Gumminess and chewiness also gradually decreased during storage, suggesting that the fruit tissue softened to some extent. However, the values of gumminess and chewiness in the UV-C-treated group were generally slightly higher than those in the CK during the middle and late stages of storage, indicating that UV-C treatment delayed the deterioration of fruit texture to some extent. Resilience showed only slight variation during storage, and the effect of UV-C treatment on resilience was not significant.

Soluble solid content (SSC) and titratable acidity (TA) are important indicators for evaluating fruit flavour, ripeness, and quality [56]. As shown in Figure 2E, SSC increased initially and then decreased in both the CK and UV-C-treated groups during storage. The CK reached its maximum SSC of 19.57 on day 4, whereas the UV-C-treated group reached its peak of 20.47 on day 12, which was significantly higher than that of the CK. This finding suggests that UV-C treatment delayed the peak in SSC without compromising the postharvest ripening process or soluble solid accumulation. The SSC of untreated passion fruit declined gradually from day 8 onwards and decreased to 18.00 by the end of storage. In contrast, the SSC of the UV-C-treated group remained at 18.97. These results indicate that UV-C treatment delayed the decline in SSC and helped maintain the edible quality of passion fruit during storage.

As shown in Figure 2F, TA decreased during storage in both groups; however, the TA level in UV-C-treated fruit remained higher than that in the CK. This result suggests that UV-C treatment contributed to the maintenance of organic acid levels during storage. Fruit respiration is one of the main causes of changes in organic acid content, as organic acids can be consumed as respiratory substrates during postharvest storage [57]. UV-C treatment slowed the respiration rate of passion fruit during storage, which was consistent with the subsequent respiration results. Therefore, UV-C treatment may delay the decline in TA by reducing substrate consumption during respiration. These findings indicate that UV-C treatment had a positive preservative effect on SSC and TA in passion fruit, thereby helping to maintain fruit flavour and edible quality during storage.

**Figure 3:**
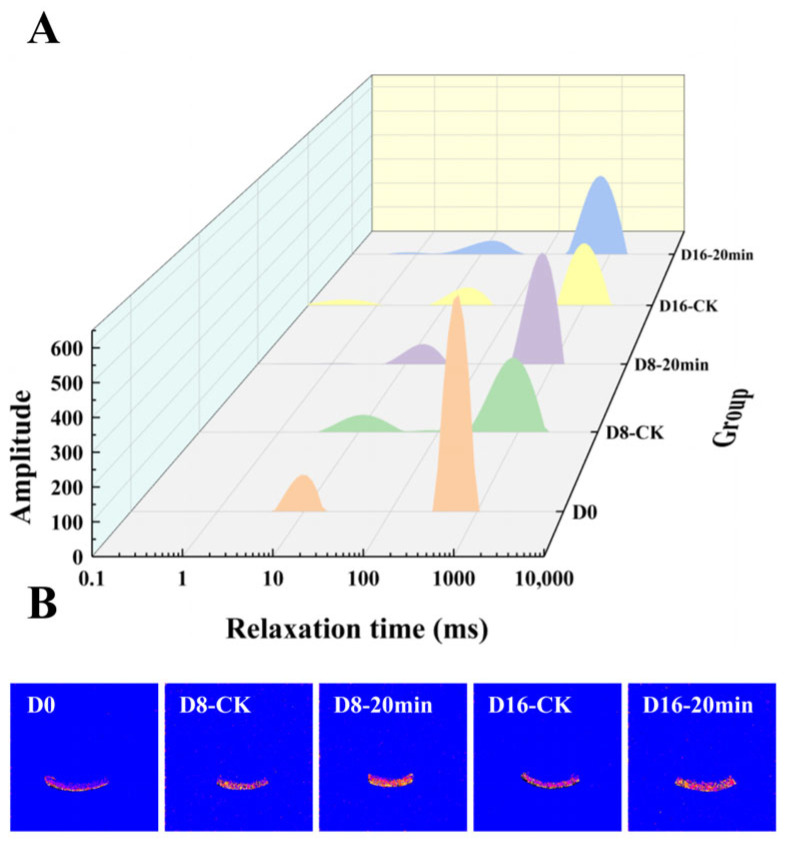
Effects of UV-C treatment on water distribution of passion fruit during storage. CK represents untreated fruit, while the treated group was exposed to UV-C for 20 min. (**A**) Transverse relaxation time (T_2_) distribution curves obtained by low-field nuclear magnetic resonance (LF-NMR); (**B**) pseudo-colour images of water distribution in fruit tissues. Different letters indicate significant differences between treatments at the same storage time (*p* < 0.05).

Low-field nuclear magnetic resonance (LF-NMR) is a useful tool for analysing water distribution and migration characteristics in postharvest fruit tissue. As shown in Figure 3A, with increasing storage time, the T_23_ peak area in the CK gradually decreased, and the peak shifted to the right with increasing relaxation time. This suggests that the mobility and distribution of free water changed during storage, which was associated with progressive water loss and peel shrivelling. This change was consistent with the progressive water loss and shrivelling observed during fruit storage [58]. On day 0, the P_23_ fraction accounted for 81.883% of the total water content of fresh passion fruit, indicating that free water was the dominant form of water present. As the fruit was stored, the P_23_ value in the CK group decreased more rapidly, dropping to 70.995% by day 8. By contrast, the P_23_ value in the UV-C treated group remained at 80.705%, suggesting that UV-C treatment helped maintain a higher proportion of free water during the early and middle stages of storage. Meanwhile, the increase in P_22_ observed in the CK group indicated a redistribution of water components during storage; this change was less pronounced in the UV-C treated group. In contrast, the decrease in the T_23_ peak area during storage was smaller in the UV-C-treated group. By day 16, the T_23_ peak area in the UV-C-treated group remained at 88.07% of that on day 0, whereas that in the CK decreased to 57.27%. This finding indicates that UV-C treatment helped maintain a relatively stable distribution of free water within the fruit and inhibited outward water migration. Furthermore, the T_22_ peak in the UV-C-treated group changed gradually, suggesting that cellular structural integrity was maintained to some extent. These results suggest that UV-C treatment helped maintain a more stable water distribution in passion fruit during storage, which may be associated with delayed tissue deterioration and reduced water loss.

As shown in Figure 3B, proton density pseudo-colour maps of the CK and 20 min UV-C-treated passion fruit were obtained during storage. The colour transition from blue to red in the LF-NMR images indicates an increase in hydrogen proton density, corresponding to differences in tissue water content. As storage time increased, the red region in the passion fruit peel gradually intensified, indicating changes in the water status of peel tissue. These changes may reflect the redistribution of water within peel tissues during storage. Compared with the CK, the UV-C-treated group showed a similar overall trend in colour changes; however, the red-stained area was slightly smaller, and the intensity was slightly lower. This suggests that UV-C treatment delayed water migration in the peel to some extent. Zhu et al. [59] reported that UV-C treatment slowed the migration of free water and reduced water loss in white fungus (*Naematelia aurantialba*), thereby helping to maintain tissue structural stability. These findings are consistent with the water migration trends observed in the present study.

### 3.3. Effects of Treatment on Fruit Respiration Rate and Ethylene Production Rate

**Figure 4:**
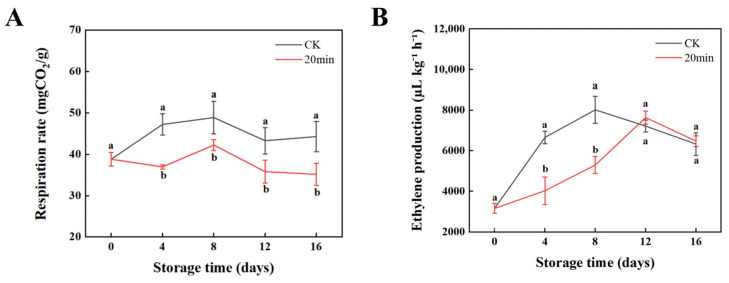
Effects of UV-C treatment on respiration metabolism of passion fruit during storage. CK represents untreated fruit, while the treated group was exposed to UV-C for 20 min. (**A**) Respiration rate; (**B**) ethylene production rate. Error bars represent the standard deviation of the mean. Different letters indicate significant differences between treatments at the same storage time (*p* < 0.05).

As a climacteric fruit, the postharvest respiration rate of passion fruit follows a pattern of initially rising and then declining. As shown in Figure 4A, the respiration rate of the UV-C-treated group remained significantly lower than that of the CK during storage. In the CK, the respiration rate exhibited a marked increase between days 0 and 8 of storage, reaching a peak on day 8. This finding suggests that the fruit had entered the ripening stage, which was supported by observable indicators such as fruit appearance and water loss-induced shrivelling. Conversely, the respiration rate in the UV-C-treated group exhibited a slight decline during the initial 4 days; although a respiratory peak was also observed on day 8, the peak value was significantly lower than that of the CK. The findings of the present study demonstrate that UV-C treatment effectively suppressed the increase in the postharvest respiration rate of passion fruit, thereby reducing the intensity of the respiratory climacteric. This, in turn, contributes to the maintenance of physiological metabolic stability and quality during fruit storage.

During the storage period, the ethylene production rate of passion fruit displayed the typical characteristics of climacteric fruit, with an initial increase in ethylene production, followed by a subsequent decrease as storage time progressed. As shown in Figure 4B, during the 0–8 day period, the ethylene production rate in the UV-C-treated group was significantly lower than that in the CK, with the difference being particularly pronounced during the early storage phase. In the CK, the ethylene production rate exhibited a marked increase during the 0–8 day storage period, rising from an initial level of 3161.19 µL kg^−1^ h^−1^ to a peak of 8018.33 µL kg^−1^ h^−1^ on day 8. In the later stages of storage, the fruits in the CK began to show marked shrivelling and browning, and the ethylene production rate gradually decreased. In contrast, the ethylene production rate in the UV-C-treated group was significantly suppressed during the early storage period, increasing to only 5299.88 µL kg^−1^ h^−1^ by day 8, which was significantly lower than the level observed in the CK at the same time point. Despite the treated group also displaying an ethylene surge during storage, the peak ethylene level was significantly reduced compared with that of the CK. Subsequently, the ethylene production rate in the UV-C-treated group reached a comparatively high level of 7636.15 µL kg^−1^ h^−1^ on day 12, followed by a slight decline in the subsequent stages of storage. These results indicate that UV-C treatment reduced the early ethylene burst and delayed the peak of ethylene production, which was consistent with the delayed changes in fruit appearance, shrivelling, and sensory quality.

### 3.4. Measurement of AsA, Total Phenolic Content, and Total Flavonoid Content

**Figure 5:**
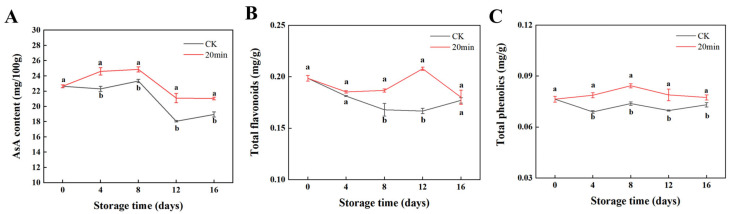
Effects of UV-C treatment on antioxidant compounds of passion fruit during storage. CK represents untreated fruit, while the treated group was exposed to UV-C for 20 min. (**A**) Ascorbic acid content; (**B**) total flavonoid content; (**C**) total phenolic content. Error bars represent the standard deviation of the mean. Different letters indicate significant differences between treatments at the same storage time (*p* < 0.05).

AsA is a significant indicator of the nutritional value of fruit and reflects its antioxidant capacity [60]. Furthermore, as a key antioxidant, AsA can directly neutralise various ROS and act as a substrate for APX in the catalytic conversion of H_2_O_2_. As shown in Figure 5A, the AsA content in passion fruit from all experimental groups exhibited a general trend of an initial increase followed by a subsequent decrease, with the CK showing a more rapid decline. During the storage period, a significant difference was observed between the CK and the UV-C-treated group. The UV-C-treated group maintained higher AsA levels than the CK, with levels remaining at 0.078 and 0.073 mg g^−1^, respectively, on day 16.

As vital secondary metabolites in the postharvest stage of fruits and vegetables, flavonoids and phenolics can effectively scavenge ROS and enhance the antioxidant capacity of plants [19]. As shown in Figure 5B, the total flavonoid content of passion fruit showed a general downward trend during storage. Specifically, the total flavonoid content in the CK decreased from day 0 to day 8 and reached a minimum of 0.17 mg g^−1^ on day 12. In contrast, the total flavonoid content in UV-C-treated passion fruit showed a significant upward trend during the middle and late stages of storage, reaching a maximum of 0.21 mg g^−1^ on day 12. This value was significantly higher than that of untreated fruit at the same stage. By the end of storage, no significant difference was observed between the two groups; however, the flavonoid content in the UV-C-treated group remained higher than that in the CK. These results indicate that UV-C treatment inhibited the decline in total flavonoid content during the early storage period and promoted flavonoid accumulation during the middle and late storage periods.

The CK exhibited a higher degree of browning in passion fruit, which may be attributed to the accumulation of brown compounds resulting from polyphenol oxidation during storage. As shown in Figure 5C, phenolic compounds, as secondary metabolites, not only confer antioxidant capacity to plant tissues but also participate in postharvest disease defence and the delay of fruit quality deterioration [61]. The total phenolic content in the CK showed an overall fluctuating downward trend, whereas that in the UV-C-treated group increased initially and then declined. During storage, the total phenolic content in the UV-C-treated group remained higher than that in the CK. The UV-C-treated group reached a maximum of 0.0843 mg g^−1^ on day 8, which was 1.14 times that of the CK. The higher levels of ascorbic acid (AsA) and total phenolic content observed in the UV-C-treated group may be associated with slower physiological deterioration of passion fruit during storage, as evidenced by a reduced respiratory rate, less oxidative damage, and delayed browning of the peel. Previous studies have confirmed that UV-C treatment preserves phenolic compound levels in various fruits [62,63]. This phenomenon can be explained by the ‘hormesis’ theory, whereby moderate UV-C irradiation activates the defence responses of fruit and vegetables [64]. In this study, preservation of phenolic compound content was consistent with higher antioxidant capacity and lower malondialdehyde (MDA) accumulation in UV-C-treated fruits. This suggests that these non-enzymatic antioxidants enhance the fruits’ oxidative stability during storage. A similar phenomenon has been observed in studies on grapes, where UV-C treatment promoted the accumulation of phenolic compounds during postharvest storage [65].

### 3.5. H_2_O_2_ Content and MDA Content

During the postharvest stage, the fruit remains in a state of high metabolic activity, and hydrogen peroxide (H_2_O_2_) within the fruit plays a dual physiological role. At moderate levels, H_2_O_2_ acts as a signalling molecule involved in the regulation of ripening and defence responses; however, its excessive accumulation induces oxidative stress, leading to membrane lipid peroxidation and damage to cellular structures, thereby accelerating fruit senescence and quality deterioration [29]. As the fruit matures and senesces, intracellular ROS homeostasis gradually becomes imbalanced, with H_2_O_2_ continuing to accumulate in the tissues. Excessive ROS production can trigger membrane lipid peroxidation, thus disrupting the structural integrity of cell membranes and ultimately leading to the formation of lipid peroxidation products such as MDA [66].

**Figure 6:**
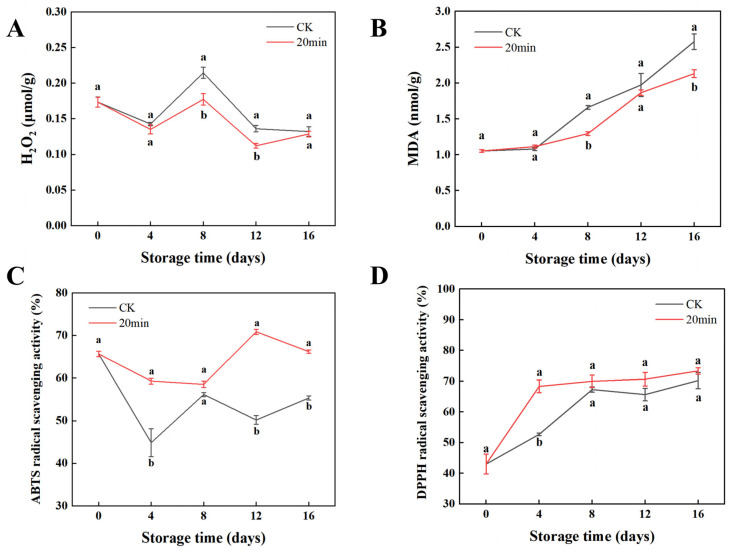
Effects of UV-C treatment on oxidative stress and antioxidant capacity of passion fruit during storage. CK represents untreated fruit, while the treated group was exposed to UV-C for 20 min. (**A**) Hydrogen peroxide (H_2_O_2_) content; (**B**) Malondialdehyde (MDA) content; (**C**) ABTS radical scavenging activity; (**D**) DPPH radical scavenging activity. Error bars represent the standard deviation of the mean. Different letters indicate significant differences between the CK and UV-C-treated groups at the same storage time (*p* < 0.05).

As shown in Figure 6A, the H_2_O_2_ content in the fruit gradually increased during storage, coinciding with the progression of fruit ripening and senescence. In the initial stage of storage, the H_2_O_2_ content in the UV-C-treated group exhibited a substantial increase; this phenomenon may be attributable to UV-C-induced oxidative stress during the early stage of storage [67]. However, during the middle and late stages of storage, UV-C treatment limited H_2_O_2_ accumulation compared with the CK, suggesting an enhanced capacity for ROS regulation. As shown in Figure 6B, the MDA content in UV-C-treated passion fruit remained lower than that in the CK during the middle and late stages of storage; moreover, by the end of storage, the MDA content in the UV-C-treated group was only 82.71% of that in the CK. This phenomenon may be attributed to the capacity of UV-C to stimulate antioxidant enzyme activity and the accumulation of non-enzymatic antioxidants within the fruit. With increasing storage time, these antioxidant systems improved the fruit’s capacity to scavenge ROS. Similar results have been reported in a range of fruits, including bananas [68], pepino [24], and citrus [69]. This suggests that UV-C treatment can regulate MDA accumulation and maintain it at a relatively low level during the later stage of storage. Concurrently, UV-C treatment suppressed lipid peroxidation in the peel of passion fruit. In the case of passion fruit, a reduction in MDA accumulation is closely linked to a decrease in membrane damage. This helps maintain the fruit’s commercial value by preserving its appearance, firmness and moisture content during storage.

### 3.6. Antioxidant Capacity: ABTS Radical Scavenging Activity and DPPH Radical Scavenging Activity

The antioxidant capacity of fruit is an important indicator for evaluating the nutritional quality of postharvest fruit [70]. Changes in ABTS and DPPH radical scavenging activities are considered to reflect alterations in the antioxidant capacity of postharvest fruit, which is closely related to reactive oxygen species metabolism and the progression of ripening and quality deterioration during storage. As shown in Figure 6C, ABTS radical scavenging activity in the CK showed a fluctuating downward trend during storage, whereas UV-C-treated passion fruit exhibited a peak value of 70.87% on day 12. This value was 41.24% higher than that of the CK. Although the ABTS scavenging rate in the UV-C-treated group declined towards the end of storage, it remained higher than that of the CK at the late storage stage, with a significant difference observed at the corresponding sampling time shown in Figure 6C.

As shown in Figure 6D, DPPH radical scavenging activity in UV-C-treated fruit increased markedly on day 4 of storage, rising from an initial value of 42.99% to 68.31%, which was 1.24 times that of the CK at the same time point. After day 8, no significant difference in DPPH scavenging activity was observed between the CK and UV-C-treated fruit. These results suggest that UV-C treatment may help to maintain antioxidant capacity. This change is associated with the metabolism of phenolic compounds within the fruit [71], and UV-C treatment may contribute to the accumulation of phenolic compounds in passion fruit, thereby potentially enhancing DPPH scavenging capacity. Similarly, UV-C treatment enhanced the overall antioxidant capacity of passion fruit during storage.

### 3.7. Antioxidant Enzyme Activities

**Figure 7:**
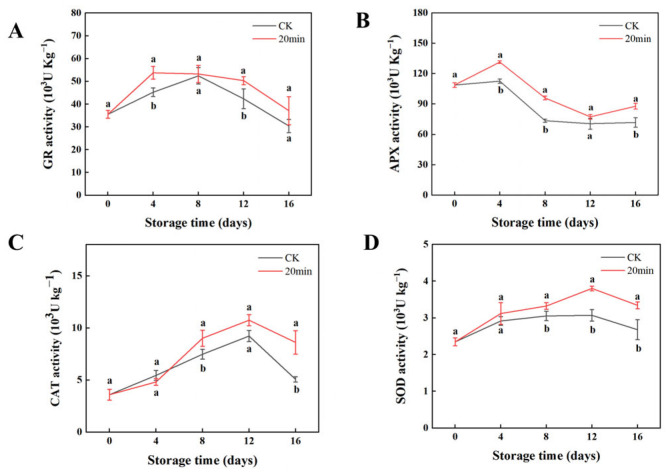
Effects of UV-C treatment on antioxidant enzyme activities of passion fruit during storage. CK represents untreated fruit, while the treated group was exposed to UV-C for 20 min. (**A**) GR activity; (**B**) APX activity; (**C**) CAT activity; (**D**) SOD activity. Error bars represent the standard deviation of the mean. Different letters indicate significant differences among treatments according to the least significant difference (LSD) test (*p* < 0.05).

The antioxidant defence system in plants comprises various antioxidant enzymes, such as SOD, CAT, APX, and GR, which scavenge excess ROS and maintain redox homeostasis within the fruit [72]. SOD catalyses the dismutation of superoxide anions (O_2_•^−^) into H_2_O_2_ and oxygen, thereby reducing the damage caused by superoxide radicals to cells. Figure 7A–D show the changes in antioxidant enzyme activities in passion fruit during storage. The present study demonstrated that SOD activity increased under UV-C treatment, with the greatest difference observed from day 8 to day 16 after treatment. During storage, SOD activity in passion fruit reached its maximum on day 12, showing an increase of 26.22% compared with the CK. UV-C treatment also resulted in a rapid increase in CAT activity, particularly during the later stages of storage (days 12–16), with the treated group exhibiting higher activity levels. SOD and CAT can scavenge superoxide anions and H_2_O_2_ within the fruit, thereby mitigating lipid peroxidation-induced damage to cell membranes, which is closely related to the maintenance of fruit firmness. The sustained increase in APX activity, together with the elevated CAT activity observed in the UV-C-treated group, may have inhibited the excessive accumulation of H_2_O_2_, which may explain the significant differences in H_2_O_2_ content between the two groups on days 8 and 12. APX uses AsA as an electron donor to reduce H_2_O_2_ within the AsA–glutathione cycle, representing an important pathway for intracellular H_2_O_2_ scavenging. APX and GR are key enzymes involved in the AsA–GSH cycle. In the UV-C-treated group, APX activity increased significantly on day 4, followed by a trend in which GR activity was also higher than that of the CK. This synergistic effect indicates that UV-C treatment effectively maintained intracellular redox balance. Changes in total antioxidant capacity, ROS-related metabolites and antioxidant enzyme activities show that UV-C treatment helps to maintain a redox balance in passion fruit during storage. The higher ABTS and DPPH radical scavenging capacities observed in the UV-C-treated group suggest an enhanced non-enzymatic antioxidant capacity, which is probably linked to higher levels of AsA, total phenolics, and total flavonoids being maintained. Meanwhile, the elevated activities of SOD, CAT, APX and GR likely contribute to the scavenging of excess ROS, particularly H_2_O_2_. Consistent with this, the UV-C-treated group exhibited lower MDA accumulation during the later stages of storage, indicating reduced membrane lipid peroxidation damage. Therefore, the alleviation of oxidative damage in passion fruit by UV-C treatment appears to result from the combined action of antioxidant substances and enzymes rather than a single antioxidant pathway.

Previous studies have demonstrated that UV-C treatment allows antioxidant enzymes to play a key role in plant responses to oxidative stress and abiotic stress, as well as in the regulation of cellular homeostasis through synergistic interactions. This, in turn, helps alleviate ROS-mediated lipid peroxidation and membrane damage [73,74].

### 3.8. Correlation Analysis and Principal Component Analysis

**Figure 8:**
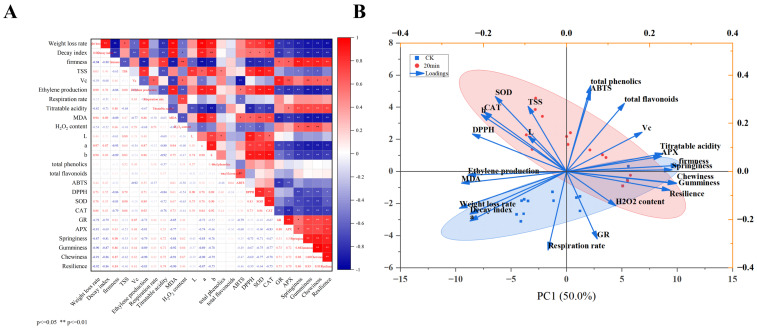
Correlation analysis and principal component analysis (PCA) of quality attributes of passion fruit during storage. (**A**) Correlation heatmap, * *p* ≤ 0.05, ** *p* ≤ 0.01; (**B**) Principal component analysis (PCA). Colour intensity indicates the magnitude and direction of correlation coefficients.

Pearson correlation analysis [75] was utilised to examine the relationships among the quality parameters of passion fruit subjected to UV-C treatment during storage (Figure 8A). The findings demonstrated a significant positive correlation between changes in the a* value of fruit colour and DPPH, SOD, and CAT, while showing a significant negative correlation with GR and APX. This indicates a correlation between changes in fruit colour and antioxidant enzyme activity. No significant correlation was observed between fruit weight loss and total phenolic or total flavonoid content. This suggests that weight loss was likely driven more by water loss, respiration and changes in tissue structure than by direct links to total phenolic or flavonoid levels under the conditions of this study. Furthermore, the shrinkage index was positively correlated with ethylene production rate and MDA content, but negatively correlated with firmness. This indicates that fruit shrinkage is closely associated with ethylene release, membrane lipid peroxidation, and tissue softening. These findings are consistent with those observed in the UV-C treated group, which exhibited lower ethylene production rate and MDA content and higher firmness.

The study also used principal component analysis (PCA) to identify changes in quality attributes, physiological parameters, and antioxidant-related indicators during storage. As illustrated in Figure 8B, the first two principal components explained 68.1% of the total variance, with PC1 and PC2 explaining 50.0% and 18.1%, respectively. Samples from different storage periods were primarily distributed along the PC1 axis, indicating that storage duration was the main factor influencing changes in passion fruit quality. Furthermore, distinct distribution patterns were observed between the control group (CK) and the UV-C-treated group, suggesting that UV-C treatment altered the overall trajectory of postharvest quality changes in the fruit. Load analysis revealed that the ethylene production rate, MDA content, weight loss rate and shrinkage index were positively correlated with PC1, while firmness, titratable acidity and APX activity were negatively correlated with PC1. These results suggest that samples with higher PC1 scores generally exhibited more severe ageing, greater water loss and more pronounced oxidative damage. In contrast, samples from the UV-C treatment group exhibited higher firmness, better acidity retention and stronger antioxidant capacity during storage. Consequently, the PCA results support the conclusion that UV-C treatment can delay the deterioration of passion fruit quality after harvest. However, the underlying regulatory mechanisms still require further validation through molecular, biological or metabolomic approaches.

### 3.9. Sensory Analysis

**Figure 9:**
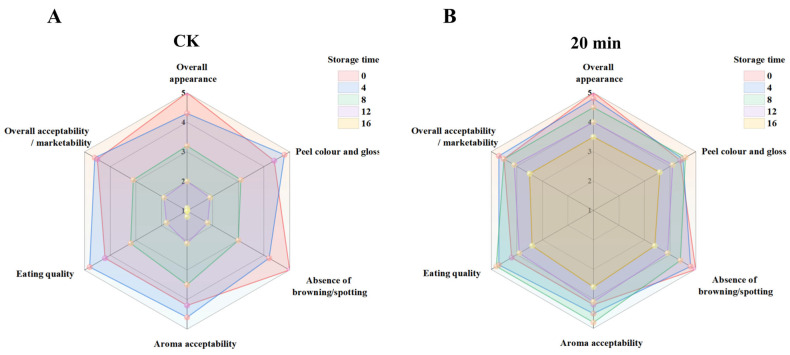
Changes in sensory quality of passion fruit during storage. (**A**) CK; (**B**) fruit treated with UV-C for 20 min. The radar plots show the scores for overall appearance, peel colour and gloss, absence of browning/spotting, aroma acceptability, eating quality, and overall acceptability/marketability at different storage times. Different colours indicate storage times of 0, 4, 8, 12, and 16 d. All sensory attributes were scored on a 5-point scale, with higher scores indicating better sensory quality.

During storage, the changes in sensory quality of passion fruit were generally consistent with the postharvest ripening and senescence characteristics of climacteric fruit (Figure 9). In the CK group, fruit ripened rapidly after harvest, with aroma acceptability and eating quality reaching relatively high scores on day 4, followed by a gradual decline during prolonged storage. After day 8, overall appearance, peel colour and gloss, and absence of browning/spotting decreased markedly in the CK fruit. The overall acceptability/marketability score decreased from 4.6 on day 4 to 3.1 on day 8 and further declined to 1.9 on day 12, indicating that CK fruit largely lost commercial acceptability between 8 and 12 d of storage.

In contrast, the UV-C-treated fruit maintained higher sensory scores throughout storage, particularly for overall appearance, peel colour and gloss, absence of browning/spotting, aroma acceptability, and eating quality. Aroma acceptability and eating quality in the UV-C-treated fruit reached relatively high levels on day 8, suggesting that normal postharvest ripening was not markedly inhibited, while sensory deterioration was delayed. By day 16, the overall acceptability/marketability score of the UV-C-treated fruit remained at 3.5, which was higher than the commercial acceptability threshold of 3.0. Based on the criterion that fruit with an overall acceptability/marketability score ≥ 3.0 was considered commercially acceptable, CK fruit maintained marketable quality for approximately 8 d, whereas UV-C-treated fruit remained commercially acceptable until 16 d. At key storage points, the UV-C-treated fruit maintained higher sensory scores than the CK. In particular, significant differences in overall acceptability/marketability were observed on day 8 and day 16, indicating that UV-C treatment helped preserve the sensory quality of passion fruit during storage. Thus, UV-C treatment extended the marketable shelf life of passion fruit by approximately 8 d under the present storage conditions.

## 4. Conclusions

In this study, 20 min UV-C treatment, corresponding to 3.6 kJ m^−2^, was selected based on preliminary screening of weight loss, shrivelling index, and appearance quality. Under the present storage conditions, UV-C treatment can significantly improve the postharvest quality of passion fruit, exerting beneficial effects on both its commercial and nutritional value. As passion fruit gradually ripens and undergoes quality deterioration during storage through the respiratory climacteric, UV-C treatment effectively slowed the fruit’s respiration rate and ethylene production rate, thereby delaying postharvest deterioration. Concurrently, UV-C treatment did not impede normal fruit maturation or yellowing, while maintaining relatively high levels of soluble solids and titratable acidity. It also enhanced the activities of antioxidant-related enzymes and promoted the accumulation of secondary metabolites within the fruit. Consequently, when an appropriate UV-C dose is applied, this method has the potential to mitigate shrivelling during passion fruit storage. This, in turn, may help maintain the commercial and nutritional value of the fruit and increase the content of bioactive compounds.

Whilst the present study confirmed the efficacy of UV-C treatment in delaying postharvest quality deterioration in passion fruit, further refinement is required regarding both its mechanism of action and practical application. From a mechanistic perspective, further elucidation is required to clarify the antioxidant response and regulation of secondary metabolism induced by UV-C using transcriptomic and metabolomic approaches. This is particularly important for understanding the regulatory mechanisms of phenylpropanoid metabolism, the AsA-GSH cycle, and ethylene synthesis pathways. Concurrently, the efficacy of UV-C treatment showed clear dose dependency, with different treatment parameters eliciting different responses in the fruit. Consequently, a more stable dose–response relationship must be established to achieve precise regulation.

In practical postharvest handling systems, the following factors should be further considered: treatment uniformity, fruit surface exposure, operational safety, equipment cost, and compatibility with existing sorting, packaging, and storage procedures. Therefore, the configuration of UV-C equipment and its treatment parameters require optimisation under pilot-scale or commercial handling conditions. Moreover, in practical applications, the stability of UV-C treatment under complex storage and transport conditions may be limited; future efforts could combine UV-C treatment with technologies such as edible coatings and modified atmosphere packaging to enhance preservation efficacy. Furthermore, its impact on flavour quality should be considered, and comprehensive assessments should be conducted through a combination of volatile compound analysis and sensory evaluation. Finally, to facilitate the practical implementation of UV-C treatment, equipment and process parameters should be optimised, and cost and safety assessments should be conducted to enhance its feasibility for the postharvest preservation of tropical fruits.

## Figures and Tables

**Table 1:** Effect of ultraviolet irradiation on the texture of passion fruit. Different letters indicate significant differences between treatments at the same storage time (*p* < 0.05).

Storage Time/Day	Springiness/mm (CK)	Springiness/mm (20 min)	Gumminess/N (CK)	Gumminess/N (20 min)	Chewiness/N (CK)	Chewiness/N (20 min)	Resilience/N (CK)	Resilience/N (20 min)
0	0.88 ± 0.03 ^a^	0.88 ± 0.03 ^a^	76.94 ± 11.09 ^a^	76.94 ± 11.09 ^a^	68.08 ± 10.77 ^a^	68.08 ± 10.77 ^a^	0.38 ± 0.03 ^a^	0.38 ± 0.03 ^a^
4	0.86 ± 0.01 ^a^	0.86 ± 0.01 ^a^	67.35 ± 1.56 ^a^	66.74 ± 16.52 ^a^	57.97 ± 1.54 ^a^	57.32 ± 14.85 ^a^	0.36 ± 0.01 ^a^	0.39 ± 0.02 ^a^
8	0.84 ± 0.01 ^a^	0.85 ± 0.01 ^a^	26.49 ± 1.58 ^b^	51.99 ± 5.23 ^a^	42.17 ± 0.96 ^a^	43.97 ± 4.73 ^a^	0.34 ± 0.01 ^a^	0.33 ± 0.01 ^a^
12	0.8 ± 0.01 ^b^	0.82 ± 0.01 ^a^	15.67 ± 3.42 ^b^	26.96 ± 11.3 ^a^	21.15 ± 1.35 ^a^	22.02 ± 9 ^a^	0.3 ± 0.01 ^a^	0.3 ± 0.01 ^a^
16	0.8 ± 0.01 ^a^	0.8 ± 0.02 ^a^	15.67 ± 3.42 ^a^	17.21 ± 5.24 ^a^	12.53 ± 2.77 ^a^	13.84 ± 4.17 ^a^	0.31 ± 0.01 ^a^	0.27 ± 0.02 ^a^

## Data Availability

The original contributions presented in this study are included in the article. Further inquiries can be directed to the corresponding authors.
